# Photoacoustic imaging of human coronary atherosclerosis in two spectral bands^☆^

**DOI:** 10.1016/j.pacs.2013.11.003

**Published:** 2013-12-05

**Authors:** Krista Jansen, Min Wu, Antonius F.W. van der Steen, Gijs van Soest

**Affiliations:** a Department of Biomedical Engineering, Erasmus MC, P.O. Box 2040, 3000 CA Rotterdam, The Netherlands; b Interuniversity Cardiology Institute of The Netherlands – Netherlands Heart Institute, P.O. Box 19258, 3501 DG Utrecht, The Netherlands; c Department of Imaging Science and Technology, Delft University of Technology, Lorentzweg 1, 2628 CJ Delft, The Netherlands

**Keywords:** Intravascular imaging, Atherosclerosis, Vulnerable plaque, Tissue characterization, Spectroscopy, Lipids

## Abstract

Spectroscopic intravascular photoacoustic imaging (sIVPA) has shown promise to detect and distinguish lipids in atherosclerotic plaques. sIVPA generally utilizes one of the two high absorption bands in the lipid absorption spectrum at 1.2 μm and 1.7 μm. Specific absorption signatures of various lipid compounds within the bands in either wavelength range can potentially be used to differentiate between plaque lipids and peri-adventitial lipids. With the aim to quantify any differences between the two bands, we performed combined sIVPA imaging in both absorption bands on a vessel phantom and an atherosclerotic human coronary artery ex vivo. Lipid detection in a human atherosclerotic lesion with sIVPA required lower pulse energy at 1.7 μm than at 1.2 μm (0.4 mJ versus 1.2 mJ). The imaging depth was twice as large at 1.2 μm compared to 1.7 μm. Adequate differentiation between plaque and peri-adventitial lipids was achieved at 1.2 μm only.

## 1 Introduction

Myocardial infarction is a leading cause of death worldwide [1]. In the majority of cases, they are caused by the rupture of an atherosclerotic plaque and the subsequent release of its thrombogenic content into the bloodstream [2]. The presence of a lipid rich necrotic core is one of the determinants of the susceptibility of a plaque to rupture [3], [4]. For that reason, the identification of necrotic core is a highly coveted imaging target. Intravascular ultrasound (IVUS) radiofrequency data analysis techniques for tissue characterization (VH-IVUS, iMap) have been developed, but their accuracy and mutual consistency are still under investigation [5], [6], [7]. Near infrared spectroscopy (NIRS) in combination with IVUS, can identify the presence but not the amount or location, relative to the lumen, of the lipid core [8], [9], [10].

**Fig. 1:**
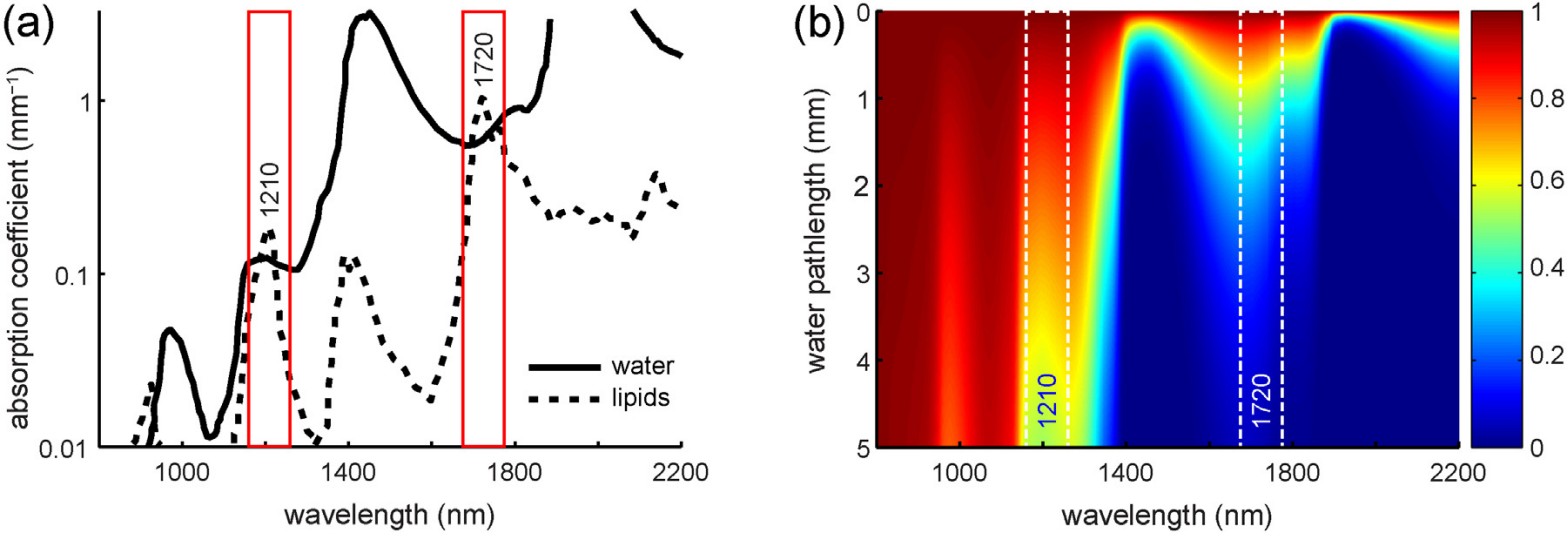
(a) Lipid and water absorption in the near-infrared wavelength region, showing the two high peaks in the lipid absorption spectrum around 1210 and 1720 nm. In these two optical windows, lipid absorption exceeds water absorption. Lipid absorption at 1720 nm is 5.5 times higher than at 1210 nm; water absorption is 5 times higher. Adapted from [22]. (b) Transmission of light through water; computed based on data from http://omlc.ogi.edu/spectra/water/abs/index.html.

Intravascular photoacoustic (IVPA) imaging has demonstrated the ability to directly image tissue components in the vessel wall, with high chemical specificity for lipid type. It utilizes differences in the absorption spectra of the vessel wall constituents to identify tissue types. Efforts have primarily concentrated on lipid detection, and started in the visible wavelength range. With the introduction of suitable light sources, focus shifted to the near-infrared wavelength range, where hemoglobin absorption is much lower, allowing for better light penetration. In this wavelength range, the absorption spectra of lipids are characterized by two prominent features around 1.2 and 1.7 μm. These absorption bands are the result of the second and first overtones of the C—H bond vibrations within the lipid molecules, respectively. The 1.2 μm absorption band has been exploited extensively to distinguish lipids from healthy vessel wall, in rabbit [11], [12] as well as human [13], [14], [15] atherosclerotic arteries. In recent years, lipid detection using excitation wavelengths around 1.7 μm has seen increased interest [13], [16], [17], [18]. In this wavelength range, the higher lipid absorption possibly leads to increased sensitivity using lower light intensity. However, water absorption is higher too, which could potentially offset the increased sensitivity for lipids by limiting the penetration depth (Fig. 1).

Both absorption bands each consist of several overlapping peaks as a result of C–H bond vibrations within the different structural groups (—CH_3_, >CH_2_, ≡CH and >CH (aromatic)) of the lipid molecules [19], [20], [21]. The position and relative height of the peaks vary with the number and location of these different structural groups within the molecules and therefore provide chemical specificity. The possibility for differentiating between plaque lipids and peri-adventitial lipids, based on the specific absorption signature of the various lipid compounds, remains to be explored.

These considerations outline tradeoffs in terms of sensitivity, imaging depth, and possibly chemical specificity connected to the choice of IVPA wavelength. In this paper, we present spectroscopic IVPA (sIVPA) imaging of a lipid containing vessel phantom and an atherosclerotic human coronary artery ex vivo at 1.2 μm and 1.7 μm, providing a direct comparison between the wavelength ranges. In the phantom, we acquired high-resolution spectra of cholesterol, cholesterol oleate and cholesterol linoleate, representative of plaque lipids, and peri-adventitial tissue. The resulting spectra were used to determine a limited number of wavelengths that maximize the difference between plaque and peri-adventitial lipids. At these wavelengths, we obtained co-registered sIVPA/IVUS images of the vessel phantom that we used to detect the plaque and peri-adventitial lipids alternatively. With two wavelengths per spectral range only, the lipid detection capability in each range was examined using both the phantom data and the ex vivo data of a diseased human coronary artery specimen.

## 2 Methods

### 2.1 Phantom design

To determine the capacity for lipid detection and differentiation at 1.2 and 1.7 μm, we made a cylindrical vessel mimicking phantom (Fig. 1a). The phantom consisted of 10% (by weight) poly-vinyl-alcohol (PVA) crystals in demineralized water that formed an acoustically transparent gel after 2 freeze/thaw cycles. It had a central lumen with a diameter of 3 mm and four 5 mm deep cylindrical cavities with a diameter of 1.5 mm, located at 500 μm from the lumen. We filled three cavities with cholesterol, cholesterol oleate and cholesterol linoleate (Sigma Aldrich Co., C8667, C9253 and C0289, resp.). These are the three most abundantly present lipids in atherosclerotic lesions [23], [24], and are assumed to be representative of plaque lipids. The fourth cavity was filled with peri-adventitial tissue that was obtained from a human coronary artery specimen, see description below. In peri-adventitial tissue, lipids are deposited as a mixture of fatty acids [25].

### 2.2 Human artery acquisition and handling

A human coronary artery was collected at autopsy from the Department of Pathology of the Erasmus Medical Center (MC), after obtaining consent from the relatives and approval of the research protocol by the Medical Ethics Committee of the Erasmus MC (MEC-2007-081). The coronary artery was frozen within 4 h at −80 °C and stored. It was thawed and measured three months later.

### 2.3 Combined intravascular photoacoustic and ultrasound imaging system

All co-registered sIVPA/IVUS images were acquired using a combined IVPA/IVUS imaging system described previously [14]. The excitation light for photoacoustic imaging was supplied by a tunable laser (OPOTEK Vibrant B/355-II) with a pulse duration of 5 ns and a repetition rate of 10 Hz. The laser was coupled to the custom-built catheter by a tapered multimode fiber (Oxford Electronics, Four Marks, UK; input diameter 1 mm; output diameter 360 μm).

The hybrid IVPA/IVUS catheter prototype we used is similar to those used earlier [14], but with a different transducer. It comprised a 400 μm diameter core optical fiber (Pioneer Optics, Bloomfield, CT) to deliver the light pulses to the vessel wall. The fiber tip was polished under a 34° angle covered by a quartz cap to maintain an air–glass interface deflecting the beam by total reflection. An ultrasound transducer was placed distal from the fiber tip to transmit and receive ultrasound waves. The 0.4 by 0.4 mm lead magnesium niobate-lead titanate (PMN-PT) single crystal ultrasound transducer was designed and custom built by the Department of Biomedical Engineering of the University of Southern California [26] and had a center frequency of 44.5 MHz and a −6 dB fractional bandwidth of 45%. The separation between fiber tip and transducer center was approximately 1 mm; the optical and acoustical beam overlapped between 0.5 and 4.5 mm from the transducer, with an angle of 22°. The catheter tip assembly had an outer diameter of 1 mm.

The catheter was rotated using a motorized rotary stage (Steinmeyer GmbH & Co. KG). For pulse echo imaging, an arbitrary waveform generator (Tabor Electronics WW2571A) transmitted a Gaussian-modulated cosine wave which was transmitted to the probe through a custom-built expander and limiter. Received US and PA signals were band pass filtered (13–60 MHz 5th order Butterworth, custom built), amplified by a 43 dB amplifier (Miteq AU1263) and digitized at a sample frequency of 350 MS s^−1^ by a 12-bit data acquisition card (Acqiris DP310).

### 2.4 Phantom measurements

Using the dual modality imaging system described above, we imaged the lipid containing vessel phantom in a water bath, with the combined IVPA/IVUS catheter positioned in the lumen. We first acquired a cross sectional IVUS image to locate the lipid inclusions. In the direction of each inclusion, we acquired sIVPA data from 1125 to 1275 nm and from 1620 to 1780 nm in steps of 2 nm, to determine the PA spectra of each lipid compound. At every wavelength, 32 image lines were recorded. The average pulse energy at the catheter tip was 1.2 mJ in the 1.2 μm and 0.4 mJ in the 1.7 μm spectral range. By staying within a relatively narrow spectral window, the average laser pulse energy and tissue scattering properties could be assumed constant within each wavelength range. The resulting spectra were normalized to the peak value and analyzed to determine the wavelengths most suitable to distinguish plaque from other lipids: per wavelength range, 4 wavelengths for which the cholesterol spectrum differed most from the spectrum of peri-adventitial lipids were selected, after which 2 wavelengths were added to create a fairly distributed spacing.

Next, we obtained two-dimensional spatially co-registered spectroscopic IVPA and IVUS images of the phantom at these 12 wavelengths (6 per wavelength range) by rotating the catheter in 1° steps and acquiring photoacoustic and ultrasound image lines at every step. At every angle, the laser was tuned through the spectral range of interest (1.2 and 1.7 μm) to ensure co-registration of the IVPA data at all wavelengths. For ultrasound pulse echo imaging, we transmitted a 10 V peak to peak Gaussian-modulated cosine wave with a center frequency of 44.5 MHz and a 50% −6 dB bandwidth relative to the peak. IVUS images were obtained by averaging the echoes from 8 transmissions per line; IVPA was not averaged (one laser pulse per wavelength per image line).

### 2.5 Artery measurements

The human coronary artery was placed in a TPX (TPX^®^ Polymethylpentene) holder with 200 μm thick metal wires glued at every 1.5 mm perpendicular to the longitudinal axis to provide image registration. The holder was then placed in a water tank containing a saline solution at room temperature. The artery was tied on a cannula through which the catheter was introduced. To find sites of interest, we performed an IVUS pullback using a commercial IVUS system (Boston Scientific iLab, Atlantis SR Pro catheters), using the metal wires as reference points. The selected wires were then found using our combined IVPA/IVUS catheter.

Spatially co-registered sIVPA/IVUS cross sectional images were obtained by rotation of the catheter in 1° steps and acquiring photoacoustic and ultrasound image lines at every step. Two rotations were performed to obtain two co-registered images at 1205 and 1235 nm, and at 1680 and 1710 nm, separately. At every angle, the laser was tuned from 1205 to 1235 nm in the first measurement, and from 1680 to 1710 nm in the second measurement, to ensure co-registration of the IVPA data per wavelength range. The ultrasound signal transmitted for pulse echo imaging, as well as the number of averaging and the average pulse energy, were the same as in the phantom measurement.

### 2.6 IVPA spectral data processing

The digitized spectroscopic IVPA data of the four lipid inclusions in the phantom, were band pass filtered between 10 and 70 MHz using a 100th order zero-phase forward and reverse finite impulse response (FIR) filter, and subsequently upsampled, corrected for jitter and downsampled to the original sampling frequency. Next, a Tukey window and envelope filter were applied. A correction for variations in the light energy was employed, using the amplitude of the signal close to the transducer, which is caused by the absorption of laser pulses in the ultrasound transducer and catheter tip. Depth locations of high signal intensity were chosen by selection of all peaks above a certain threshold in the 1205 and 1710 nm enveloped signal traces, respectively. Spectra at selected locations were filtered using a fourth order digital smoothing polynomial (Savitzky–Golay) filter, 32 times averaged and normalized.

### 2.7 sIVPA/IVUS image reconstruction

The digitized IVPA and IVUS rotational data band pass filtered and jitter corrected like the spectroscopic scans described above. The 1.2 μm wavelength range IVPA data of the artery cross section where subsequently median filtered over 5 image lines in the angular direction for extra noise reduction.

An adaptive filter was designed and applied to all rotational IVPA data to remove the artifact that was caused by the absorption of laser pulses in the ultrasound transducer and catheter tip. It presents in the IVPA image as bright rings, concealing the photoacoustic signals produced by the arterial tissue close to the catheter. A similar circular artifact in the IVUS data, caused by the ‘ringing’ of the transducer as a result of the transmission of ultrasound pulses, was removed by subtracting the mean in the angular direction of the affected part of the data. Both the IVPA and IVUS data were then Tukey windowed and envelope filtered. A correction for variations in the light energy between individual pulses and between the different wavelengths, using the amplitude in the ring artifact mentioned above, was applied to the IVPA data. We subsequently scan-converted the IVPA and IVUS data to Cartesian coordinates and log compressed them for display. The ‘hot’ and ‘gray’ colormaps in Matlab (R2007b) were used for the IVPA and IVUS images, respectively. To create combined IVPA/IVUS images, we overlaid the IVPA data on the IVUS images using a nonlinear red-yellow-white color scale and a linear transparency scale. All data processing was done using Matlab (R2007b).

### 2.8 sIVPA data analysis for lipid differentiation and detection

**Fig. 2:**
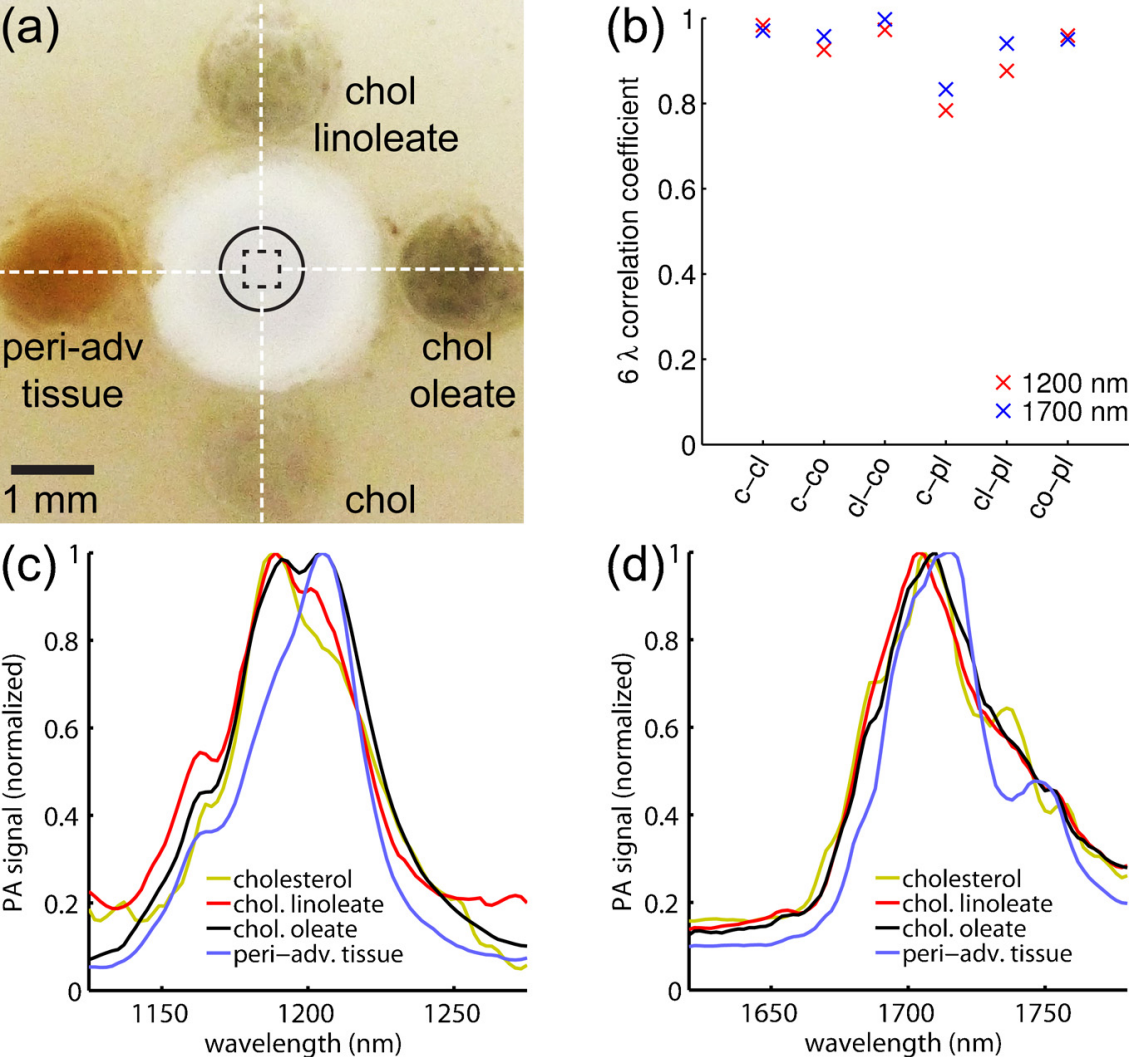
Lipid containing vessel phantom and IVPA spectra of lipid inclusions. (a) Photograph of the phantom (top-view), filled with cholesterol (bottom), cholesterol oleate (right), cholesterol linoleate (top) and peri-adventitial tissue (left). (b) 6-Wavelength correlation coefficients between the spectra of the lipid inclusions. (c) Average, normalized PA spectra of the four lipid inclusions in the 1.2 μm wavelength range, and (d) in the 1.7 μm wavelength range. c, cholesterol; cl, cholesterol linoleate; co, cholesterol oleate; pl, peri-adventitial lipids.

To investigate the capability of sIVPA in the two absorption bands to distinguish plaque from peri-adventitial lipids, the two 6-wavelength sIVPA data sets of the lipid containing vessel phantom were processed as described above, up to scan-conversion. For each pixel in the resulting data sets, the correlation coefficient *R* of the PA spectrum with two reference spectra was computed. We used the PA spectra of cholesterol and peri-advential tissue as reference spectra for plaque and peri-adventitial lipids, respectively. Of the plaque lipid spectra (Fig. 2c and d), in either spectral range, the cholesterol spectrum has the lowest correlation with the peri-adventitial spectrum (Fig. 2b) and therefore is the most suitable to distinguish plaque from other lipids.

The 6-wavelength correlation coefficients *R*_*x*_ (*x* is lipid type; “chol” for cholesterol or “PL” for peri-adventitial lipids) were median filtered over 4° in the angular direction and 8 samples in the radial direction. The threshold values *R*_c,*x*_ for the correlation coefficients were chosen empirically: the lowest values for which plaque lipids could still be separated from peri-adventitial lipids were selected. To create a lipid map, the lipid matching regions–those with a correlation coefficient equal to or higher than the threshold value *R*_c,*x*_ – were displayed in red and overlaid on the corresponding IVUS image.

We compared the potential of sIVPA at 1.2 μm and at 1.7 μm for lipid identification using two wavelengths, *λ*_*h*,*ω*_ and *λ*_*l*,*ω*_ (*h* for high lipid absorption; *l* for low lipid absorption) per wavelength range *ω*. We computed lipid maps of the phantom and artery cross section using the following algorithm: we determined the noise level at *λ*_*h*,*ω*_ by sampling the PA signal inside the lumen (identified in the IVUS image) and masked out all PA signal below that level. We then calculated the relative difference *δ*_*ω*_ = [*I*(*λ*_*h*,*ω*_) − *I*(*λ*_*l*,*ω*_)]/*I*(*λ*_*hω*_), where *I*(*λ*) is the PA signal amplitude at wavelength *λ*. *δ* was subsequently median filtered over 4° angular by 8 samples radial. Lipids were identified by *δ* > *δ*_*c*_, where *δ*_*c*_ are the threshold values determined from the analysis of the absorption spectra of pure lipids, see Fig. 2, Fig. 5.

### 2.9 Histological validation

After imaging, we cut the artery at the two wires adjacent to the imaging plane to obtain a 3 mm thick artery segment with the imaged cross-section in the middle. The segment was embedded in “optimal cutting temperature” (OCT) compound (Tissue-Tek^®^, Sakura Finetek Europe B.V.), frozen in liquid nitrogen cooled isopentane vapor, and stored at −80 °C until serial sectioning for staining. We performed Oil Red O (ORO) staining to identify lipids (stained red). A Hematoxylin–Eosin (H&E) stain was used to provide an overview of the artery cross section; a Resorcin–Fuchsin (RF) stain was used to demonstrate the morphology and fibrous structure of the vessel cross-sections.

## 3 Results

### 3.1 Lipid differentiation in phantom

We performed sIVPA measurements in the directions indicated by the white dashed lines in the photograph (top view) of the phantom in Fig. 2a. The data were analyzed as described in Section 2.6 and the resulting averaged normalized PA spectra of cholesterol, cholesterol oleate, cholesterol linoleate and peri-adventitial tissue in the 1.2 and 1.7 μm wavelength range are shown in Fig. 2c and d, respectively. While the spectra at 1.2 μm exhibit mainly differences in relative peak height, the dominant differences at 1.7 μm are shifts in the locations of the peaks between the spectra of plaque and peri-adventitial lipids.

**Fig. 3:**
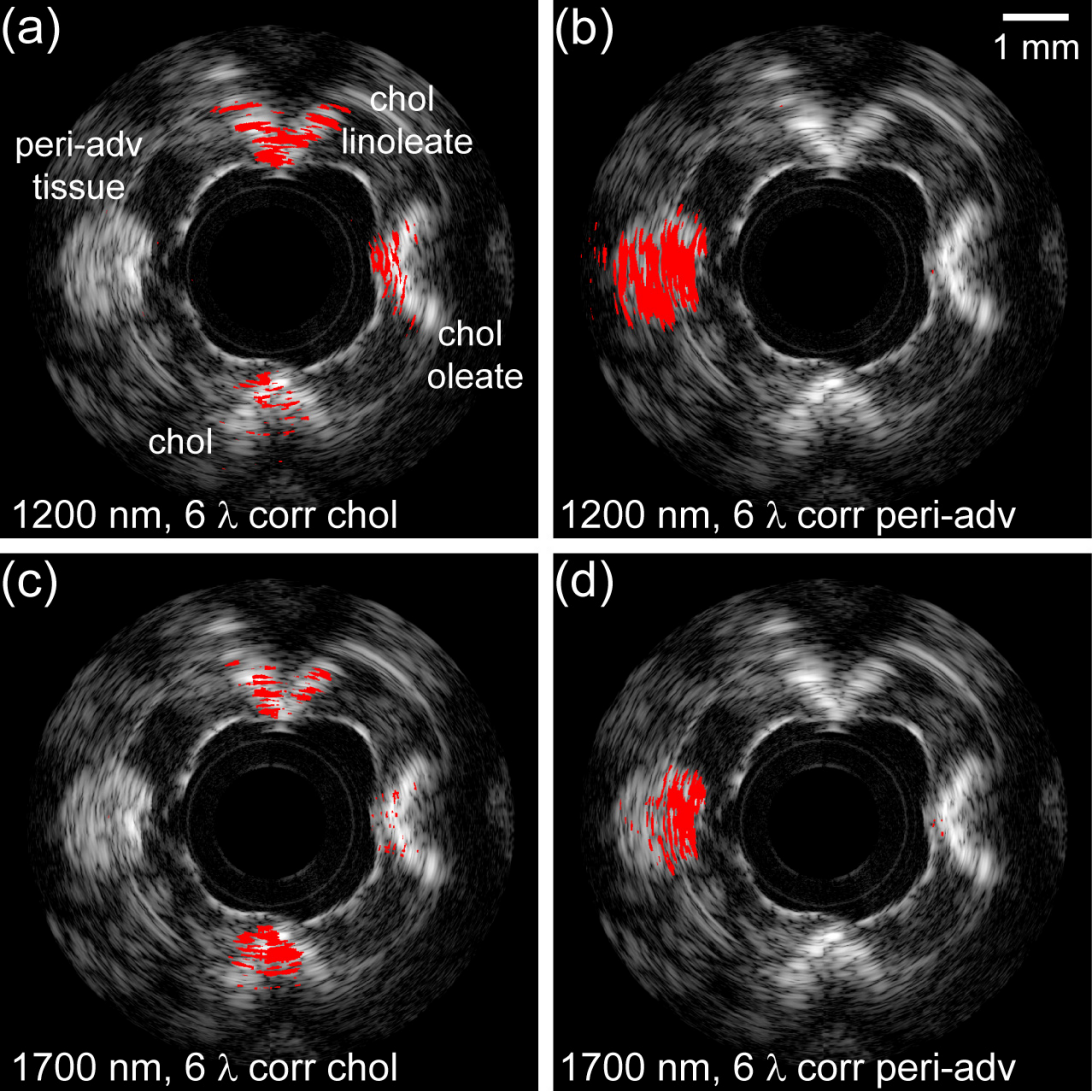
Lipid typing in a lipid-containing vessel phantom using sIVPA at 1.2 and 1.7 μm. (a) Lipid map based on 6-wavelength correlation with the cholesterol, and (b) with the peri-adventitial reference spectrum in the 1.2 μm wavelength range (1185, 1195, 1205, 1215, 1225 and 1235 nm). (c) Lipid map based on the 6-wavelength correlation with the cholesterol, and (d) with the peri-adventitial reference spectrum in the 1.7 μm wavelength range (1680, 1710, 1718, 1726, 1734 and 1751 nm). All lipid maps are shown overlaid on the corresponding IVUS image (dynamic range 65 dB). Plaque lipids, represented by cholesterol (bottom), cholesterol oleate (right) and cholesterol linoleate (top) are distinguished clearly from peri-adventitial tissue (left) at 1.2 μm, while lipid typing at 1.7 μm yielded an inferior result.

We obtained cross sectional sIVPA/IVUS data of the lipid-containing vessel phantom at 6 wavelengths in both spectral ranges. The wavelengths are given in Table 1. The lipid maps resulting from the correlation of the data in the 1.2 μm range with the cholesterol and the peri-adventitial lipid reference spectrum are displayed in Fig. 3a and b, respectively; The corresponding lipid maps obtained in the 1.7 μm range are shown in Fig. 3c and d, respectively. All lipid maps are overlaid on the associated, co-registered IVUS image. At 1.2 μm, the cholesterol and the two cholesterol esters, representative of plaque lipids, are all detected clearly, while the peri-adventitial tissue remained concealed (Fig. 3a). At 1.7 μm, however, it was not possible to simultaneously detect the cholesterol oleate and keep the peri-adventitial tissue invisible. On the other hand, more of the cholesterol was detected than at 1.2 μm. The correlation with the peri-adventitial reference spectrum resulted in detection of the peri-adventitial tissue in the 1.2 μm wavelength range, while suppressing the other lipids (Fig. 3b). In the 1.7 μm wavelength range, less of the peri-adventitial tissue was detected (Fig. 3d).

**Table 1:** Wavelengths used for 6-wavelength lipid detection.

Wavelength range (μm)	Wavelengths (nm)
1.2	1185, 1195, 1205, 1215, 1225, 1235
1.7	1680, 1710, 1718, 1726, 1734, 1751

Table 2 lists the threshold correlation values *R*_c,*x*_ for the 6-wavelengths at 1.2 and 1.7 μm that were used to create the lipid maps in Fig. 3a–d. To be able to detect peri-adventitial lipids while suppressing plaque lipids, the correlation coefficients had to be chosen much higher for the 1.7 than for the 1.2 μm wavelength range.

**Table 2:** Threshold values *R*_c,*x*_ and *δ*_c_.

Threshold variable	Wavelength range (μm)	
	1.2	1.7
*R*_c,*chol*_	0.87	0.88
*R*_c,*PL*_	0.88	0.96
*δ*_c_	0.37	0.23

### 3.2 Lipid detection in phantom and artery

**Fig. 4:**
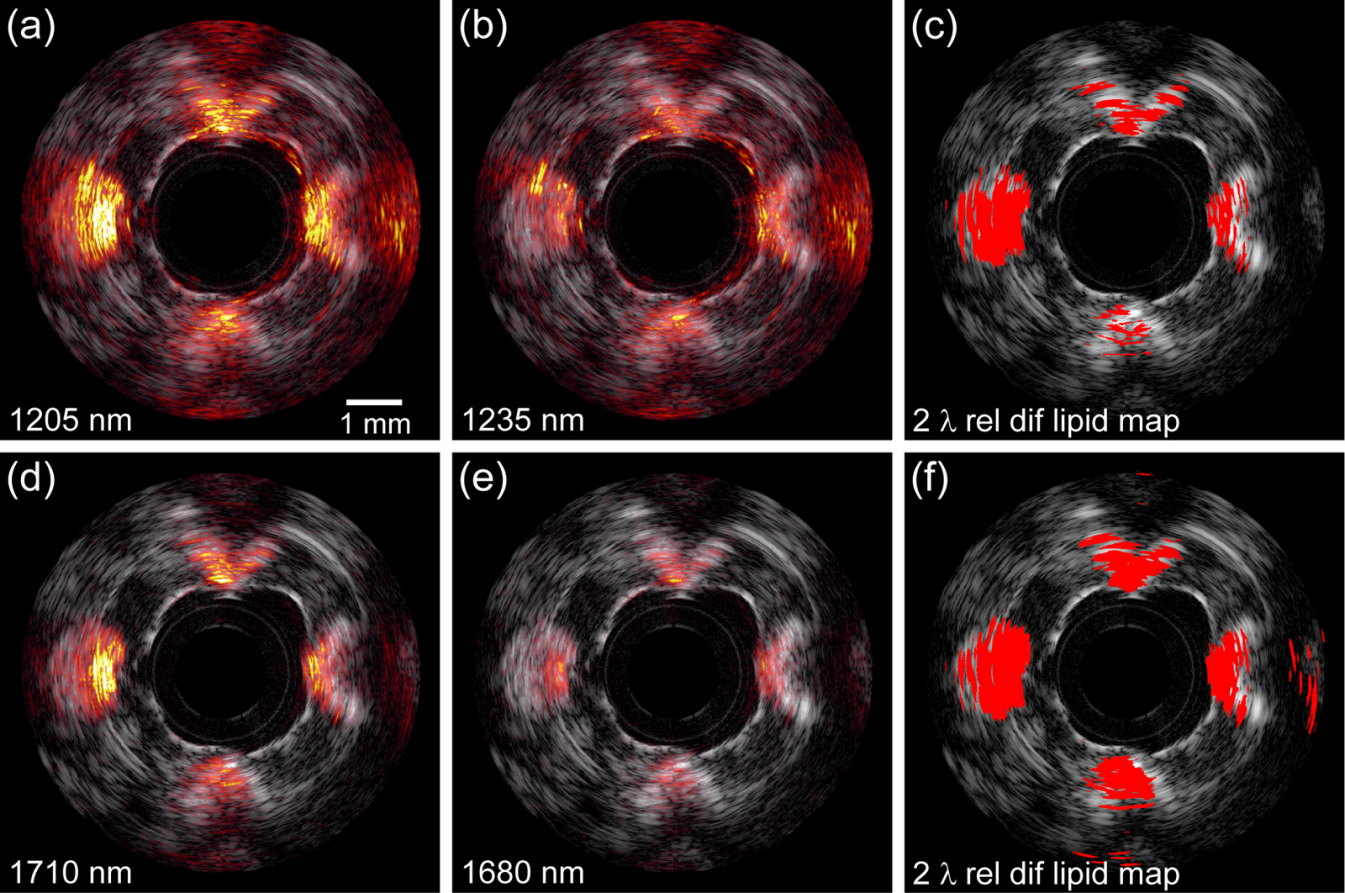
Lipid detection in a lipid-containing vessel phantom using sIVPA at 1.2 and 1.7 μm. (a) 1205 nm and (b) 1235 nm combined IVPA/IVUS images (IVPA 50 dB, IVUS 65 dB) of PVA phantom filled with cholesterol (bottom), cholesterol oleate (right), cholesterol linoleate (top) and peri-adventitial tissue (left). (c) Lipid map based on 2-wavelength relative difference between the PA signal at 1205 nm and 1235 nm. (d). Co-registered 1710 nm and (e) 1680 nm combined IVPA/IVUS images (IVPA 50 dB, IVUS 65 dB) of the same cross section of the vessel phantom. (f) Lipid map resulting from the 2-wavelength relative difference between the PA signal at 1710 nm and 1680 nm. Both lipid maps are shown overlaid on the corresponding IVUS image.

We investigated the ability of sIVPA to detect lipids using two wavelengths at 1.2 μm versus the 1.7 μm, in both the lipid containing vessel phantom and a human coronary artery ex vivo. The phantom results are shown in Fig. 4. The co-registered combined IVPA/IVUS images at 1205 (high lipid absorption) and 1235 nm (low lipid absorption) and the resulting relative difference lipid map, are displayed in Fig. 4a–c, respectively. Fig. 4d–f depicts the corresponding high and low lipid absorption images in the 1.7 μm wavelength range, at 1710 and 1680 nm, and lipid map, respectively. In both wavelength ranges, all four lipid containing cavities exhibit an increased PA signal at *λ*_*h*_ compared to the PA signal at *λ*_*l*_. In the 1.2 μm range IVPA images, however, a higher signal can be observed from non-lipid regions, compared to the 1.7 μm range IVPA images, due to the overall higher light fluence. Both lipid maps succeed equally well in displaying the peri-adventitial lipid region but the plaque lipids were displayed more clearly in the 1.7 μm wavelength range.

The false positives in the 1.7 μm lipid map at 2 times the distance of the lipids from the catheter are the result of incomplete suppression of the pulse echo signal that is generated by absorption of the light in the transducer and catheter tip. This ultrasound signal is also visible in the 1.2 μm range but is effectively suppressed, as well as the higher PA background signal.

**Fig. 5:**
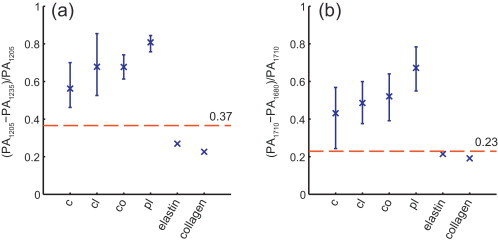
Relative difference of PA signal at high and low lipid absorption wavelengths of individual (unaveraged) IVPA spectra measured in the lipid containing phantom. (a) 5th to 95th percentile of the relative difference of the 1205 nm and 1235 nm PA signal strength of cholesterol, cholesterol oleate, cholesterol linoleate and peri-adventitial lipids (32, 128, 160 and 96 spectra, respectively). Data for elastin and collagen are obtained from [25]. (b) 5th to 95th percentile of the relative difference of the 1205 nm and 1235 nm PA signal strength of same lipid components (same number of spectra). Elastin data obtained from [27]; collagen data from [28]. c, cholesterol; cl, cholesterol linoleate; co, cholesterol oleate; pl, peri-adventitial lipids.

The threshold values *δ*_*c*_ to realize the lipid maps in Fig. 4c and f, were chosen using the spectral data of the separate lipid compounds. The 5th to 95th percentile of the relative difference of the PA signal at high and low lipid absorption wavelengths of the individual (unaveraged) IVPA spectra measured in the lipid containing phantom were calculated (Fig. 5). The relative difference of the absorption coefficient of elastin and collagen at these wavelengths, is shown as well. Threshold values were chosen as the midpoint between the lowest value found for the lipids and the highest value found for elastin and collagen (red dotted line). The resulting threshold values *δ*_*c*_ in both wavelength are listed in Table 2.

**Fig. 6:**
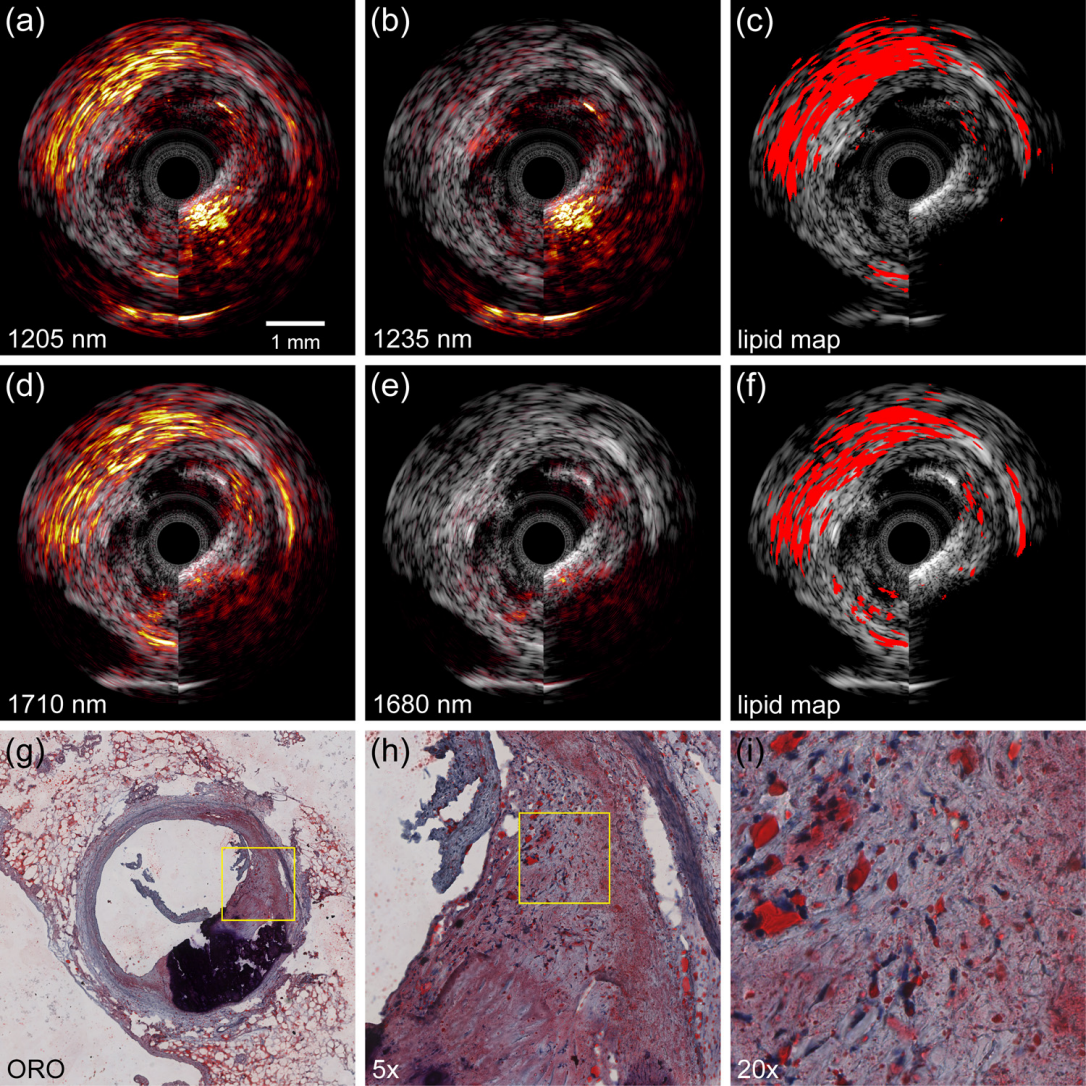
Lipid detection in an atherosclerotic human coronary artery using sIVPA at 1.2 and 1.7 μm. (a) 1205 nm and (b) 1235 nm combined IVPA/IVUS images (IVPA 25 dB, IVUS 40 dB). (c) Lipid map based on 2-wavelength relative difference between the PA signal at 1205 nm and 1235 nm. (d). 1710 nm and (e) 1680 nm combined IVPA/IVUS images (IVPA 25 dB, IVUS 40 dB). (f) Lipid map resulting from the 2-wavelength relative difference between the PA signal at 1710 nm and 1680 nm. Both lipid maps are shown overlaid on the corresponding IVUS image. (g) Lipid histology stain (ORO); lipids are stained red; calcification is stained black (h) 5× magnification of the part of the atherosclerotic plaque indicated as lipid rich by the lipid stains (area outlined in black in (g)), shows larger extracellular lipid droplets, while the lipids in all other parts of the lesion are intracellular or contained in small extracellular droplets. (i) 4× magnification of area outlined in black in (h).

The results of the atherosclerotic human coronary specimen (left anterior descending artery, male aged 65) measurement are shown in Fig. 6. The top row, Fig. 6a–c, displays the results obtained in the 1.2 μm wavelength range; the bottom row, Fig. 6d-f, the results obtained using wavelengths around 1.7 μm. From left to right, the IVPA images at *λ*_*h*_, the IVPA images at *λ*_*l*_, and the 2-wavelength relative difference lipid maps are shown. All images are overlaid on the corresponding IVUS image. The IVUS images show a small lumen with an eccentric plaque at the bottom right of the vessel wall. A large calcification is present in the plaque, indicated by the shadowing in the plaque area. The ORO lipid stain, depicted in Fig. 6g, confirms these findings (lipids in red; calcification black). The 1205 nm IVPA image shows a slightly enhanced signal from the lipid area in the top part of the plaque, as well as a strongly enhanced signal in the peri-adventitial region around the vessel wall, compared to the 1235 nm IVPA image. In the 1.7 μm range, the enhancement of the signal in the lipid rich plaque area is more pronounced than in the 1.2 μm range, while the signal enhancement in the peri-adventitial region is comparable. Additionally, the calcified region produces much less signal around 1.7 μm, albeit still in the same order of magnitude as the signal produced by the plaque lipids. In accordance with the differences found by visual inspection of the IVPA images, both lipid maps indicate lipids in the top part of the lesion, as well as in the peri-adventitial tissue region around the vessel wall. In the 1.7 μm lipid map however, more intraplaque lipids are detected, due to the higher signal enhancement and therefore better signal to noise ratio. The signal from the calcification is suppressed successfully in both cases. The enlargements of the ORO lipid stain (Fig. 6e and f) of the area indicated as lipid rich in the lipid maps reveal the presence of larger extracellular lipid droplets, whereas the lipids in all other parts of the lesion are intracellular or contained in small extracellular droplets. This preferential detection is possibly caused by a higher Grüneisen coefficient, a higher concentration of the lipids or a better matching of the generated PA signal frequency to the bandwidth of the transducer.

The data presented here (in Fig. 3, Fig. 4, Fig. 6) show that the maximum lipid imaging depth at 1.2 μm is approximately twice as large compared to 1.7 μm. The exact numerical value depends on the optical (scattering and absorption) and acoustic (frequency-dependent attenuation) properties of the tissue. At 1.2 μm, lipid signal was received from tissue layers in the artery wall at a depth of 5 mm.

## 4 Discussion and summary

This study assesses the lipid detection and distinction capabilities of spectroscopic intravascular photoacoustic imaging in two absorption bands, around 1.2 μm and 1.7 μm. We acquired co-registered sIVPA/IVUS data of a lipid containing vessel phantom at 6 wavelengths in either spectral window. Correlation with a cholesterol PA spectrum, as reference for atherosclerotic lipids, and with a peri-adventitial tissue reference spectrum, using 6 wavelengths from 1185 to 1235 nm, distinguishes very well between atherosclerotic lipids and peri-adventitial lipids. The same 6-wavelength correlation method applied on sIVPA data from 1680 to 1751 nm resulted in a poorer separation of in particular cholesterol oleate and peri-adventitial tissue. Applying a 2-wavelength relative difference method, we successfully detected all four lipid compounds present in the vessel phantom in both wavelength ranges, with a superior detection of all four lipids in the 1.7 μm region. In an ex vivo sIVPA/IVUS measurement of an human coronary artery, we found superior plaque lipid contrast in the 1.7 μm wavelength range, with a lower pulse energy (0.4 mJ versus 1.2 mJ at 1.2 μm) and sufficient imaging depth (for this particular vessel cross section). Low pulse energy is an advantage because it lowers the optical power that needs to be dissipated in vivo, and also reduces artifacts in IVPA imaging caused by light absorption in the catheter.

The relative difference between two wavelengths is a robust parameter to detect the lipids, also in the presence of strong water absorption in the longer wavelength band, as long as the signal received from the lipids is above the noise level. Note that a minimum of two wavelengths is required in both the 1.2 μm and the 1.7 μm wavelength range to distinguish lipids from other vessel wall constituents; the IVPA signal generated by calcium is in the order of magnitude of the signal from lipids around 1.7 μm and even higher around 1.2 μm.

Minimization of the required number of wavelengths is important for clinical application: the acquisition speed is inversely proportional to the number of PA acquisition needed to compose an image line. Light sources are expected to represent a significant fraction of the cost of an IVPA system for clinical applicability, presenting another reason to limit the number of wavelengths. We demonstrated here that two wavelengths are sufficient, in principle, to detect lipids, but not to differentiate. Noise reduction in the acquisition and more sophisticated analysis, targeted to exploit spectral differences between the various lipid tissues, may yield more insight from a two-wavelength combination.

This study is qualitative in design, exploring several analysis methods applied to the different lipid absorption bands for lipid detection and differentiation by sIVPA. A larger quantitative study will be performed in the future to determine the most favorable wavelengths and the appropriate parameters used for processing. Such a study could also establish whether the imaging depth at 1.7 μm is sufficient to image the vessel wall of larger coronary arteries, such as the left main stem, completely. A larger ex vivo study will also elucidate the representativeness of pure cholesterol, cholesterol oleate, and cholesterol linoleate for the absorption spectra of real atherosclerosis. It will provide insight into the natural variability of the absorption spectrum, which the eventual choice of wavelength combinations will have to take into account. Photoacoustic imaging seems to favor the detection of larger extracellular lipid droplets. To quantify the sensitivity of sIVPA to the different forms and sizes of intraplaque lipids, a statistical analysis of a larger data set should be performed.

In summary, we presented the lipid detection and typing capabilities of 1.2 μm and 1.7 μm sIVPA. We observed superior lipid differentiation in the shorter wavelength range in a lipid containing vessel phantom. In the longer wavelength range, however, intraplaque lipid detection was improved, both in the vessel phantom as well as in an atherosclerotic human coronary artery, with lower pulse energy.

## Conflict of interest statement

The authors declare that there are no conflicts of interest. The funding agency had no involvement in the study, the writing of the manuscript, or its submission.
